# Understanding spatiotemporal patterns of global forest NPP using a data-driven method based on GEE

**DOI:** 10.1371/journal.pone.0230098

**Published:** 2020-03-10

**Authors:** Siyang Yin, Wenjin Wu, Xuejing Zhao, Chen Gong, Xinwu Li, Lu Zhang

**Affiliations:** 1 Key Laboratory of Digital Earth Sciences, Aerospace Information Research Institute, Chinese Academy of Sciences, Beijing, China; 2 State Key Laboratory of Remote Sensing Science, Beijing Normal University, Beijing, China; 3 Sanya Institute of Remote Sensing, Hainan, China; Virginia Commonwealth University, UNITED STATES

## Abstract

Spatiotemporal patterns of global forest net primary productivity (NPP) are pivotal for us to understand the interaction between the climate and the terrestrial carbon cycle. In this study, we use Google Earth Engine (GEE), which is a powerful cloud platform, to study the dynamics of the global forest NPP with remote sensing and climate datasets. In contrast with traditional analyses that divide forest areas according to geographical location or climate types to retrieve general conclusions, we categorize forest regions based on their NPP levels. Nine categories of forests are obtained with the self-organizing map (SOM) method, and eight relative factors are considered in the analysis. We found that although forests can achieve higher NPP with taller, denser and more broad-leaved trees, the influence of the climate is stronger on the NPP; for the high-NPP categories, precipitation shows a weak or negative correlation with vegetation greenness, while lacking water may correspond to decrease in productivity for low-NPP categories. The low-NPP categories responded mainly to the La Niña event with an increase in the NPP, while the NPP of the high-NPP categories increased at the onset of the El Niño event and decreased soon afterwards when the warm phase of the El Niño-Southern Oscillation (ENSO) wore off. The influence of the ENSO changes correspondingly with different NPP levels, which infers that the pattern of climate oscillation and forest growth conditions have some degree of synchronization. These findings may facilitate the understanding of global forest NPP variation from a different perspective.

## Introduction

Forests play an important role in the global carbon cycle, and the net primary productivity (NPP) of forests is a vital indicator that not only relates to its ecological characteristics and regional climate but also highly depends on their ability to adapt to changes in the surroundings [1]. Many factors can affect forest NPP. Previous studies showed that high temperature and water deficit constrain the accumulation of forest NPP [2–5], and radiation-limited vegetation accounts for approximately 27% of the earth’s vegetation surface [6]. In addition, forest traits and cover densities have strong effects on forest NPP [7–10]. The relationships between NPP variation and the El Niño-Southern Oscillation (ENSO) phases have also been studied in a wide spectrum [11–14]. To obtain general conclusions about the distribution and variation of forest NPP, researchers usually divide a study area into different regions according to their geographical location or climate types [15–18], which may separate the areas that have the same NPP values into different categories and tend to conclude incomparable results by using different categorical variables. Moreover, considering that the carbon absorption of forest ecosystems has intricate interactions with different impact factors and exhibits spatiotemporal heterogeneity, further analysis from a different perspective that can exhibit the combined influence of impact factors on the NPP in a more effective way is needed to better understand the global forest NPP variation.

We are now in an era of big earth data, in which a large number of satellite and aerial data can be accessed continuously, dynamically, and freely. Due to the limitations of online transmission and computing power, it takes a lot of time to download and process a large amount of data; therefore, it is necessary to change the traditional method. Under this background, cloud remote sensing platforms such as Google Earth Engine (GEE) (https://earthengine.google.com/), Amazon Web Services (AWS) (https://aws.amazon.com/), GBDX (https://platform.digitalglobe.com/gbdx/), and Climate Engine (https://climateengine.org/) have emerged since 2014. Although there are still many gaps to fill before making these platforms are widespread in remote sensing science, they promote an entirely new way of data usage, in which downloading data is no longer needed and weeks of processing can be finished in just a few minutes thanks to the parallel computing.

In this paper we leverage the GEE platform to figure out the influence of different impact factors on forest NPP and how forests of different NPP-levels respond to climate change and short-term climate fluctuations (i.e., ENSO events), and to understand spatiotemporal dynamics of the global forest NPP and explore its relationships with forest traits, climate conditions, and ENSO. The article is organized as follows: datasets and preprocessing are introduced in the second section; methodology is depicted in the third section; results and analyses are presented in the fourth section; comparison and discussion are shown in the fifth section; and finally, we conclude the article in the last section.

## Datasets and preprocessing

To consider different aspects of forest and climate features, we selected six temporal variables (Table 1), including NPP, normalized difference vegetation index (NDVI), fraction of photosynthetically active radiation (FPAR), land surface temperature during daytime (LSTD), land surface temperature at night (LSTN), and precipitation (Rain), which all have collections on GEE in 2004–2013. We chose this period considering both the availability of data and consistency of the product quality. The first five variables are derived from the corresponding MODIS products [19–22], and for the last variable, we adopted the Global Satellite Mapping of Precipitation (GSMaP) product, which provides hourly precipitation rates based on multiband space-borne passive microwave and infrared radiometers [23–26]. The ESA GlobCover2009 product, which contains twenty-two land cover classes defined according to the United Nations land cover classification system, is employed to distinguish forest areas [27]. The MODIS land cover product is not selected because it is not available in 2013 on GEE, and GlobCover2009 provides a more detailed forest type division, which may better support the analysis. Datasets and their GEE ImageCollection IDs are shown in Table 1. Detailed information such as spatial and temporal resolution can be found via GEE explorer (https://explorer.earthengine.google.com/) by referring to the ID of each dataset. In addition, we also collected the climate zone dataset from ArcGIS (http://services.arcgis.com/BG6nSlhZSAWtExvp/) and the Oceanic Niño Index (ONI) data, which is a widely adopted representation of the ENSO phases from NOAA (http://origin.cpc.ncep.noaa.gov/products/analysis_monitoring/ensostuff/ONI_v5.php).

**Table 1:** Datasets with GEE ImageCollection IDs.

Index	Dataset	GEE ImageCollection ID
1	ESA 300 m 5-year Landcover	ESA/GLOBCOVER_L4_200901_200912_V2_3
2	MODIS 500 m Annual NPP	MODIS/006/MOD17A3H
3	MODIS 500 m 16-day NDVI	MODIS/MOD13A1
4	MODIS 500 m 4-day FPAR	MODIS/006/MCD15A3H
5	MODIS 1 km 8-day LSTD&N	MODIS/MYD11A2
6	0.1 degree Hourly Precipitation	JAXA/GPM_L3/GSMaP/v6/reanalysis

Although there are four datasets derived from MODIS data, the input data and derivation methods used in their algorithm are relatively different, which could contribute to the independence of these products to some extent. The LSTD and LSTN data are extracted from the MODIS land surface temperature/emissivity dataset, which is derived based on the MODIS level 1B calibrated and geolocated radiances of bands 31 and 32 (TIR) during the day and night, respectively. The NDVI and FPAR are generated mainly using the MODIS surface reflections of the red and NIR bands, among which the NDVI is calculated with simple ratio operation while the FPAR is derived using a 3D radiative transfer equation that further considers vegetation structure and sun-sensor geometry. While the back-up algorithm of FPAR, which is used when the main method fails to localize a solution, is based on empirical relationships between the FPAR and NDVI, there is about 11% of data used in this study may influenced by this back-up algorithm (please see the S2 Fig for the detailed information) and these data may bring uncertainties to the correlation analysis among impact factors.

Data collection and preprocessing are performed using the GEE python API on Google Cloud to leverage the powerful python statistical processing to support the analysis. First, Datasets 2–6 are collected from 2004 to 2013 with a spatial resolution of 10 km, and then datasets 3–6 are resampled into monthly time series and filtered by Dataset 1 to remove the non-forest areas. The land cover types for forest-dominated ecosystems that are used in this study are shown in Table 2. Pixels with more than half of the missing values are removed to ensure the validity of the analysis. The GEE script file can be found at https://github.com/winggy/GEE-forest-analysis. Consequently, the annual NPP with 10 collections (1 collection per year) and factors with 120 collections (12 collections per year) including the NDVI, FPAR, LSTD, LSTN, and Rain are derived. Finally, ONI statistics in 2004–2013 and climate zone data are uploaded to Google Cloud to interact with the other parameters. All of the data and process are compiled with the terms of service for the GEE.

**Table 2:** Land covers for forest-dominated ecosystems in GlobCover2009.

Index	Forest type
40	Closed to open broadleaved evergreen and/or semideciduous forest
50	Closed broadleaved deciduous forest
60	Open broadleaved deciduous forest
70	Closed needleleaved evergreen forest
90	Open needleleaved deciduous or evergreen forest
100	Closed to open mixed broadleaved and needleleaved forest
110	Mosaic Forest/Shrubland / Grassland
130	Closed to open shrubland
170	Closed broadleaved semideciduous and/or evergreen forest regularly flooded—Saline water

## Methodology

To support the data-driven analysis, forest areas are categorized using a self-organizing map (SOM) method [28] according to their NPP levels instead of climate or forest types. This method of segmentation will link all the analyses to the NPP. The SOM is a type of neural network that trains the input samples with competitive learning, which is a kind of unsupervised learning to produce a clustering map [28, 29]. Unlike some traditional methods, the SOM does not require the input data to conform to a normal or linear distribution. This method can extract the rules and features of the input data through unsupervised learning and map the multidimensional complex data to the low-dimensional space in a nonlinear manner while effectively retaining the measurement relationship and topology of the input data [30]. During learning, the neural network first computes the Euclidean distances between the input samples and weight vectors of all neurons. Then, the nearest node to the training datum is selected, and the weights of this neuron v and its neighbor u are updated as

$$W_{v}(s+1)=W_{v}(s)+\theta (u,v,s)∙\alpha (s)∙(D(t)-W_{v}(s))$$ (1)

to move toward the inputs, where W_v_ is the current weight of node v, s denotes the current iteration, θ(u, v, s) measures the distance between u and v in step s in the neighborhood, α(s) is a decreasing learning coefficient, and D(t) represents the input vector in which t is an index used to scan the training set systematically. After several iterations, the grid formed with the neural network is able to approximate the data distribution. A software library for Matlab, SOM Toolbox 2.1 (https://github.com/ilarinieminen/SOM-Toolbox), was used to obtain NPP categories in this study.

To select the number of output categories, we mainly considered two aspects. One is that the distances between NPP time series curves of the obtained clusters should be large enough to avoid largely overlapping. At the same time, the number of clusters should be as large as possible within certain limits. Therefore, we changed the output number from 5 to 20 and employed the Davies-Bouldin index (DB) to evaluate the clustering results. The Davies-Bouldin index is defined as the average similarity measure of each cluster with its most similar cluster, where similarity is the ratio of within-cluster distances to between-cluster distances [31]. Clusters that are farther apart and less dispersed will result in a lower value. The index is calculated as

$$DB=\frac{1}{k}\sum_{i=1}^{k}\frac{c_{i}+c_{j}}{d_{i,j}}$$ (2)

where, k denotes the number of clusters, c_i_ is the average distance between each point of cluster i and the centroid of this cluster and d_i,j_ is the distance between centroids of clusters i and j.

In the experiment, the result with the smallest DB were chosen, which was calculated with ten neurons, and with the node weight vectors initialized randomly to derive an arbitrarily positioned map in the data space. After the training, nine NPP-related forest categories were obtained and indexed with C1-9 according to their NPP levels from low to high, and the whole dataset was clustered (please refer to S1 Fig for the result of SOM clustering). The associated climate zones and forest types for each category were computed. Furthermore, time series of the NPP, NDVI, FPAR, LSTD, LSTN, and Rain were aggregated for each category, and the correlations between all of these parameters were measured. Finally, the variations of all these parameters were compared with the dynamics of the ENSO phases to see how forests with different NPP levels respond to climate change and the potential reasons behind those responses.

## Results and analyses

### The geographical distribution and NPP variation of different NPP-related forest categories

**Fig 1:**
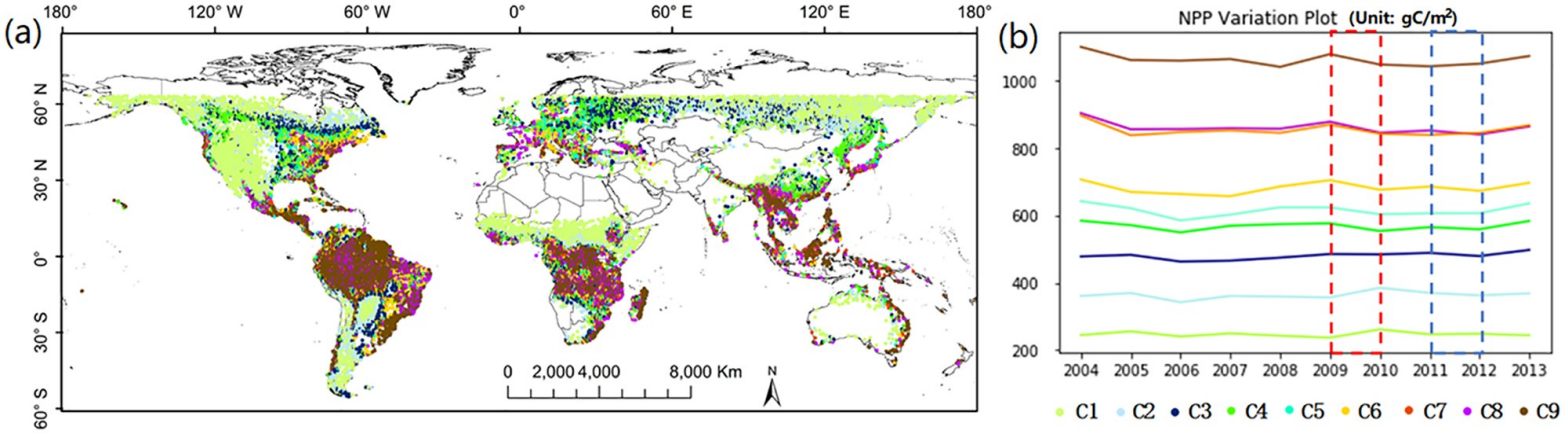
The distribution map (a) and NPP time series (b) of nine NPP-related forest categories.

Fig 1 shows the geographical distribution map and NPP time series of the nine NPP-related forest categories obtained with the SOM. On the whole, C1 and C9 account for about 50% of the whole study area, while C5 and C7 are two smallest proportion among these 9 categories. The average proportion of C2-4, C6 and C8 are about 9%. In Fig 1(a), we can observe that C1-3 are mainly located in the western North America, southwestern South America, Northeastern Asia and central Africa, where have relative poor climate conditions for forest growth. C4-5 are primarily located in eastern North America, Europe and eastern coast of Asia, where have relative humid climates. C6-8 have relative discrete distribution, scattered across the tropical area, the eastern North America, the southern Europe and the eastern Asia. C7-C9 primarily located in tropical area, where is characterized by hot and wet.

From the NPP time series figure, we can find that the major difference between these categories are their overall NPP levels, which corresponds well with our intention of clustering. An exception is the pair C7 and C8, which has similar NPP levels with slightly different variation trends during 2010–2012. Considering that the proximity of the distribution locations of these two categories, this deviation implies that their interaction mechanisms with certain environmental factors are different. Moreover, it is interesting to note that signatures of the low-NPP group, including C1 and C2, peak in 2010, while those of the high-NPP group, C6-9, reach the peak in 2009 (C6 is not a typical high-NPP categories but seems more like a transition stage from the low-NPP group to the high-NPP group). These characteristics may relate to the ENSO events in year 2009–2010. These patterns probably indicate that different NPP levels may be related to distinct response mechanisms of the forest to climate change.

### The climate conditions and forest types of different NPP-related forest categories

**Fig 2:**
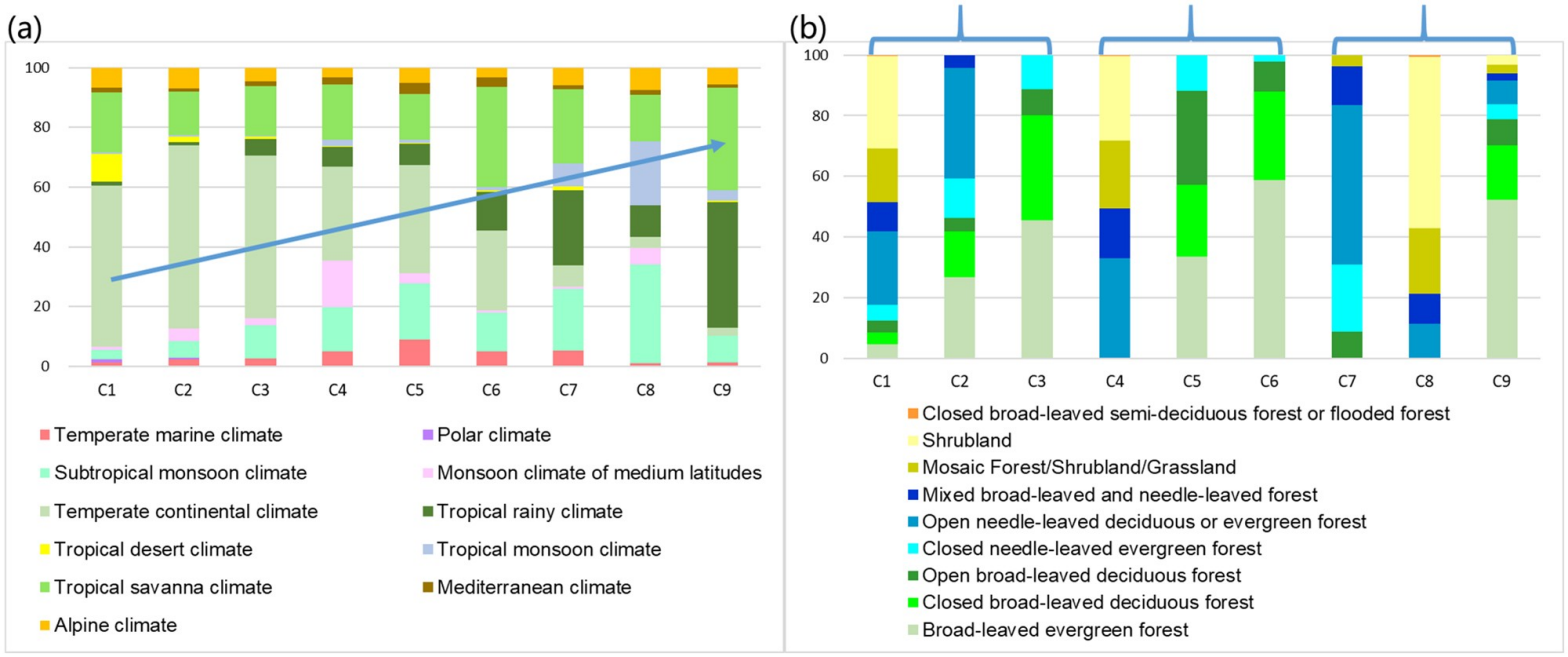
The associated climate zones (a) and forest types (b) for the NPP-related forest categories. The blue arrow in (a) indicates the increasing proportion of climates suited to forest growth.

Fig 2 shows the associated climate zones and GlobCover2009 forest types for the nine NPP-related forest categories. We can see that each of these NPP-related categories corresponds with a variety of climate zones and forest types, which provides the analysis of global forest NPP with different perspective compared with previous studies. That is, rather than perform analysis on separate climate zones and tree categories, this study performs analysis based on different NPP levels. We observe that C1, which has the lowest NPP level, contains a large portion of the tropical desert and continental climate, and its main forest types are shrubland and open needle-leaved forest. C2 has a similar composition of climate zones as C1 but it contains more broad-leaved forest, which may be the reason for the increase in the NPP compared with C1. C4 and C5 also have similar compositions of climate zones but comparing with C4, C5 has more area of tall, dense, and broad-leaved forests which cause a higher NPP of C5. C3, C6 and C9 all have a relatively large proportion of broad-leaved forest, but the gradually increase of the proportion of warmer and wetter climates makes the NPP of these three categories: C9>C6>C3. For the pair with similar NPP levels, C7 and C8 have distinct forest type components. C7 mainly corresponds to needle-leaved forest, while C8 corresponds to shrubland. As for climate types, C7 has relatively large proportion of tropical rainy and savanna climate, while C8 has more proportion of tropical and subtropical monsoon climate. These differences may inherently make C7 relative insensitive to the climate change. C9 contains the most tropical rainy climate and includes over 70% broad-leaved forest, which leads it to the highest NPP level.

Comparing the distribution of climate types and forest types of these nine categories, we can observe that with the NPP levels change from low to high, the composition of the climate zones gradually changes from cold and dry climates to warm and humid climates, while the composition of the forest types shows three similar patterns: C1-3, C4-6, and C7-9. The similarity can be more significant when switching C7 and C8, which are pairs with similar NPP levels. In each pattern, the forest type changes from shrubland with lower trees to forest with taller trees, from sparsely distributed to densely distributed, and from needle-leaved to broadleaved. This imparity of change patterns between climate and forest type indicates that compared with the traits of the forest, the climate is a more dominant impact factor for forest NPP.

### Correlations between the NDVI, FPAR, LSTD, LSTN, and Rain of different NPP-related forest categories

**Fig 3:**
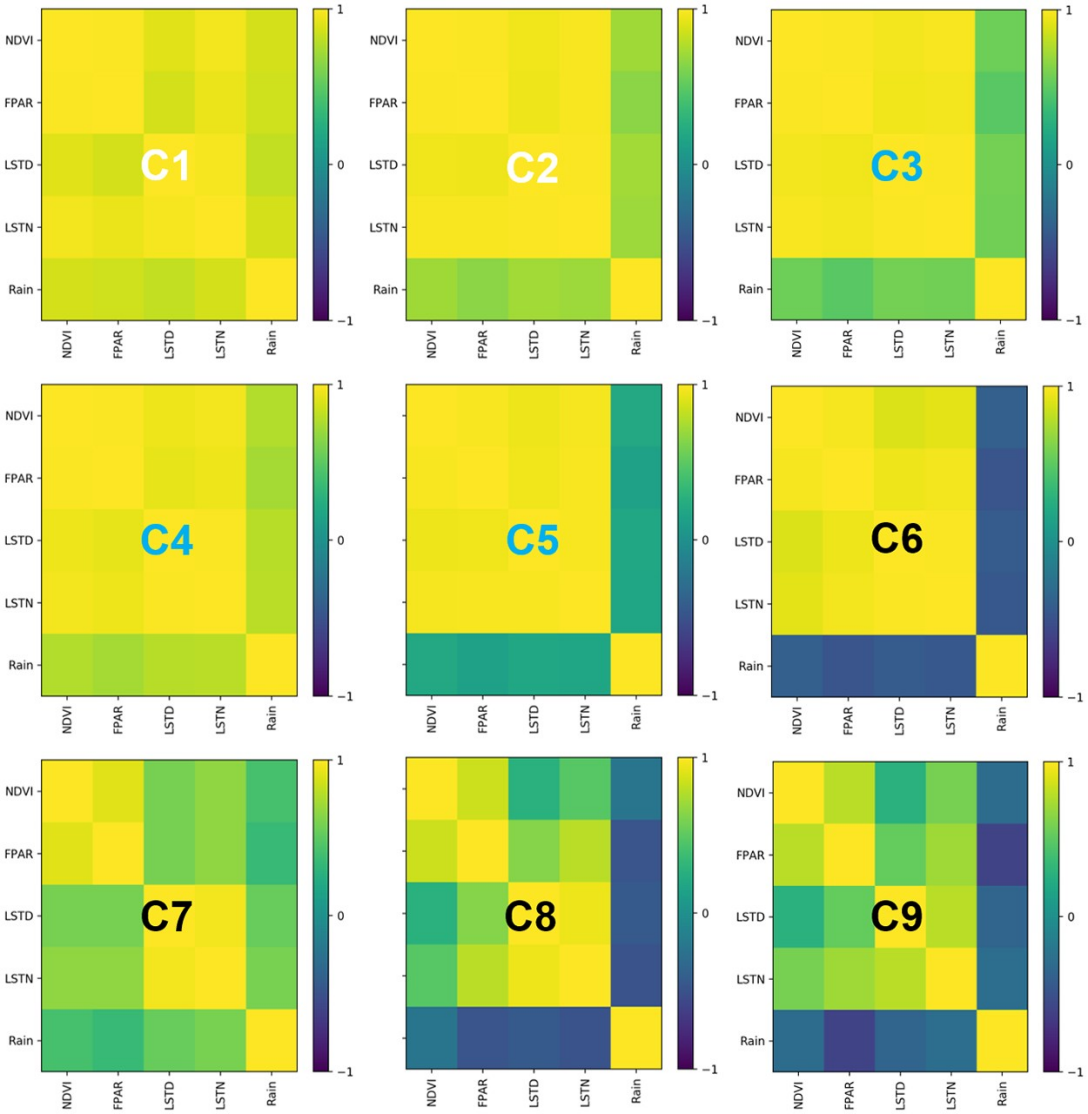
Correlations between the NDVI, FPAR, LSTD, LSTN, and Rain for each NPP-related forest category. White indexes represent NPP signatures that peaked in 2010, black ones denote NPP signatures that peaked in 2009, and cyan ones indicate the other cases.

To see how different NPP levels relate to typical forest ecological parameters and climate factors, Pearson correlation coefficients [32] are measured between each pair of selected impact factors for each NPP-related forest category. The computation is implemented on the normalized 10-year monthly time series of the NDVI, FPAR, LSTD, LSTN, and Rain, and the results are shown in Fig 3. To better show the characteristics of the inter-annual NPP variation for each category, we use white indexes to represent categories with NPP signatures that peaked in 2010, black ones to denote those with NPP signatures that peaked in 2009, and cyan ones to annotate the other cases.

Among the five factors, NDVI directly reflects the physiologically functioning greenness level of vegetation canopies [33–35], and the other four factors are used to reflect the climate status. We can see that for classes C1-4 with relatively low NPP levels, the five factors have all positive correlations with each other, and the difference only exists in the correlation strength between the precipitation and the other factors. It is interesting to note that although C3 has a larger portion of tall and broad-leaved trees compared with C4, the weaker correlation between precipitation and the other climate factors might restrict its NPP. For C5, precipitation has no linear correlation with the other factors. Recalling that C4 and C5 have similar geographical distributions and climate status but different forest types, we can infer that the difference of correlations among precipitation and other factors between these two categories may attribute to the higher proportion of broad-leaved forest in C5 which may be relatively insensitive to climate change compared to needle-leaved forest and shrubland in C4. The high-NPP categories, C8 and C9, have similar correlation patterns, in which the precipitation has weak or negative correlation with the other factors, and the LSTs has weak or positive correlation with NDVI. The forests of the two categories are mainly distributed in tropical climates which are characterized by high temperature and precipitation and are mainly limited by radiation [36]. For C6, which is in the transition stage from the low-NPP group to the high-NPP group, the precipitation has negative correlations with the other factors, while the other four factors all have positive correlations with each other. As for C7, similar with C4 and C5, C7 and C8 have proximal geographical locations and NPP levels, while the higher proportion of forests in C7 may make it relative insensitive with climate change than C8. As a result, comparing with C8, NDVI of C7 has relatively weak correlations with the LSTD, LSTN and Rain.

In general, we can see that forests in different NPP-related categories show different correlations among impact factors. With the increase of NPP levels, NDVI always has a positive correlation with FPAR, while the correlation between the NDVI and LSTs gradually decreases and the correlation between NDVI and precipitation changes from positive to negative (except C7). This implies that for forests with low NPP levels, which mainly located in areas that have lower precipitation, temperature and FPAR compared to optimal growth conditions, NDVI has strong positive correlation with all climate factors. However, for forests with high NPP levels, mainly located in tropical and subtropical areas that are characterized by hot and wet climates, NDVI has strong positive correlations with FPAR, but weak or even negative correlations with temperature and rainfall. Compared with temperature, precipitation is more likely to show negative correlations with the variation of NDVI for high NPP level forests, and this may explain why forests are mainly radiation-limited in the tropics. It is also easy to notice that the difference between the correlation maps of the categories with 2009 peak and those with 2010 peak mainly exists in the distinct influence of the precipitation, while C7 is an exception for its special forest type.

### Variation in forest ecological parameters and climate factors during ENSO events of different NPP-related forest categories

**Fig 4:**
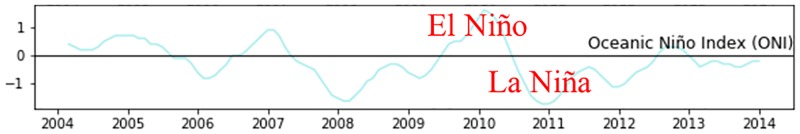
The ONI signature in 2004–2013.

The ENSO is a periodically occurring climate phenomenon that affects both the atmosphere and the ocean in tropical regions. Fig 4 shows the monthly ONI from 2004 to 2013, in which positive values indicate the warm ENSO phase (El Niño events) and negative values denote the cold ENSO phase (La Niña events). During El Niño, the sea surface temperatures (SST) in the central and eastern tropical Pacific Ocean are higher than average, and thus the rising motion of heated air increases over these regions as well as tropical Africa, which leads to heavier rainfall in these areas. During La Niña, SST in the central and eastern Pacific Ocean are cooler than average, and the surface easterly winds across the tropical Pacific are stronger. Rising air motion increases over the western Pacific and Indonesia as well as northern South America. For the Pacific Ocean has vast size, El Niño can increase global temperature, while La Niña cools the temperature [37]. Both of these events typically last approximate 9–12 months, and often begin to form during June-August, reach peak strength during December-April, and then decay during May-July of the next year. During the study period, relatively strong El Niño events occurred in 2009–2010 [38], while La Niña events were during 2007–2009 and 2010–2012. In 2009, the LA Niña of 2007–2009 was weakening and the El Niño of 2009–2010 was forming during January to September, following with the peak of El Niño from October 2009 to March 2010. From April to June 2010, El Niño gradually declined and the next La Niña gradually formed, followed by the peak of this event between July 2010 and February 2011. This La Niña subsided until June 2012 when ONI became positive. By comparing with Fig 1, we can observe that high-NPP forest categories increase the NPP at the onset period of El Niño, while low-NPP categories increase their NPP a year later. Similar but weaker phenomena can be found in 2004–2005, which is part of a prolonged El Niño event from 2002 to 2005. Note that because the ENSO phases usually maximize or minimize in the middle of a year, annual NPP, which is the accumulated statistics of a whole year, may not exactly correlate with the event (for example, the NPP in 2009 actually correlates with the La Niña of 2007–2009 and the El Niño of 2009–2010).

**Fig 5:**
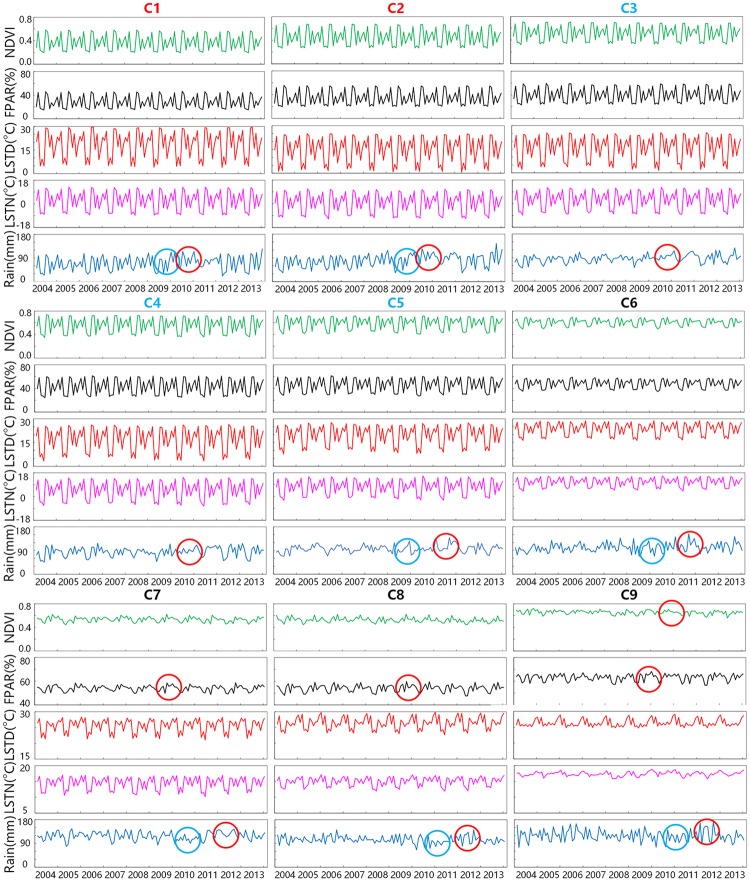
The monthly series of the NDVI, FPAR, LSTD, LSTN, and Rain. Anomalies are marked using red (increasing) and cyan (decreasing) circles for the NPP-related forest categories. Red indexes represent NPP signatures that peaked in 2010, black ones denote NPP signatures that peaked in 2009, and cyan ones indicate the other cases.

Fig 5 presents the monthly series of forest ecological and climate factors for each NPP-related forest category. The anomaly sections of the time series are marked with red and cyan circles to denote the increasing and decreasing phenomena, respectively. We can see that in most of the time period, the annual cycles of these curves are very significant, and the patterns repeat from year to year. Multiple peaks can be observed because of the combined effects of different seasonal patterns in distinct hemispheres and climate zones. We are surprised to see the obvious periodical trends of most of these temporal curves considering that the statistics are averaged all over the world and are supposed to contain numerous uncertainties.

Among the five parameters, precipitation shows the weakest periodicity and the most obvious disturbance during 2009–2012, which corresponds to one El Niño event and one La Niña event. We observe that the low-NPP categories C1-2 show relatively lower precipitation in 2009 and relative higher in 2010–2011. C1-2 primarily distributed in northwestern North America, northern Asia and north central Africa where there was lower annual rainfall during El Niño and higher rainfall during La Niña. As water is insufficient for local forests, precipitation in these regions is positive for NPP and annual NPP peaks in 2010. C3-4 have relatively higher precipitation in 2010–2011, while C5-6 have relatively lower rainfall in 2009–2010 and increased rainfall in 2011. C3-6 mainly located in eastern of North America, Europe and eastern of Asia, and precipitation of these areas tend to decrease during El Niño events. C7-C9 present relative lower precipitation in 2010–2011, while the precipitation increased during 2011–2012. Meanwhile, FPARs of these high-NPP categories are relative higher in 2009–2010 while LSTs are relative higher in 2010–2011. The forests of C7-9 categories are mainly distributed in tropical regions. During El Niño events, the Walker circulation was weakened and the western tropical Pacific became warmer and dryer, while the other way around during the La Niña. Compared to the FPAR, LST and precipitation in these regions had about half of year lag in response to ENSO events.

For high-NPP categories, the FPAR is relative higher during 2009–2010 and the precipitation and LSTs tend to about half of year lag in response to this El Niño event. In 2009, forests of these categories had sufficient water, heat and radiation, thus their NPP increased, while in 2010, precipitation decreased and LSTs increased, though there were enough radiation, the drought weather lead to a decrease of NPP. In 2011, precipitation increased and temperature decreased, but the decrease of FPAR limits the increase of NPP. This result in line with previous studies on the influence of drought on forests which found that although forests might show “green-up” in a severe drought, the annual NPP declined sharply [39–41]. For low-NPP categories, as the inter-annual variance of impact factors other than precipitation is relatively small, the variance of forest NPP of these categories is mainly affected by precipitation. The increase of precipitation in 2010 leaded to an increase in forest NPP of these low-NPP categories. Moreover, while the NPP of high-NPP categories decrease in the 2010, their NDVIs show relative slight change and the NDVI of C9 even presents an increasing trend (S3 Fig). We can infer that the role of climate factors in forest greenness and forest carbon absorption may not be entirely the same.

## Discussion

Currently, the scale and types of remote sensing data that are available have been expanding [42, 43]. It is difficult to perform a global-scale study involving long time series and multisource parameters in a traditional way which would take a lot of time and local storage space to download and process data. Therefore, we present a case study that uses the cloud platform GEE to process geospatial data online and avoid downloading thousands of images. Limited by the data accessibility of the chosen variables, we can only obtain the time period of 2004–2013 currently. However, with more data uploaded on GEE, we can perform analyses using longer time series and diverse parameters to obtain more generic conclusions and discover additional interesting phenomena. We hope that this work will provide insight into and benefit relative research.

The variation in forest NPP and forest greenness are related to various influential factors. A study by Bastos et al. analyzed the response of the NPP to heat and drought and illustrated that the strong decrease of the NPP in 2010 could not be explained solely by water stress [2]. Nemani et al. showed that water, temperature, and radiation could impose limits on vegetation growth by approximately 40%, 33%, and 27% of the global surface, respectively [6]. In this study, we show that the synchronization between water and heat has correlation with forest greenness, and forests with different NPP levels show different relations with this synchronization. Forests with low-level NPP locate in areas that have insufficient water and heat compared to optimal growth conditions, and their forest greenness shows positive relationship with both precipitation and temperature. Categories with high NPP levels are distributed in areas with abundant water, and the main restrictions on tree growth are solar radiation. Although precipitation shows negative correlation with forest greenness for the high-NPP categories, insufficient water may cause less productivity for low-NPP categories. It should be noted that there is a portion of the FPAR data in this analysis is derived from the NDVI (S2 Fig) as a backup algorithm used by the MODIS team [44], and therefore, may affect the correlation between the NDVI and FPAR In addition, although MODIS data is vulnerable to cloud contamination in the tropics, most of the information of MODIS products reflects real surface characteristics rather than data errors as this impact is minimized using overlapping observations and retrieval algorithms [45, 46]. The analysis of the percentage of fill value of yearly NPP and monthly NDVI based on the corresponding quality control data is shown in S4 Fig.

Previous studies have shown that the ENSO explains over 40% of global NPP variability, particularly for tropical and subtropical areas [11, 47]. During warm ENSO events, the net and total productivity will both decrease, while during cool ENSO events, the global terrestrial biosphere increases in carbon sink [48, 49]. In Figs 4 and 5, we show that forests with different NPP levels respond differently to ENSO events. The low-NPP forest categories respond mainly to the La Niña event and show an increase in precipitation, which simultaneously causes an increase in the NPP. The high-NPP forest categories present increased LSTs and NPP at the onset of the El Niño event, while decreased precipitation and NPP in the following year. The middle-NPP categories show a lagged increase in precipitation compared with the low-NPP categories, and their NPP shows relative slight change. The influence of ENSO on forests changes correspondingly with the NPP levels from low to high, though these categories are not clustered based on geographical locations. Therefore, we may infer that the pattern of climate oscillation and forest growth conditions have some degree of synchronization.

The impact of ENSO events on forests is an interesting topic that has been studied by many researchers. Malhi et al. summarized studies on the Amazon forest and concluded that the variance in precipitation, which was critically affected by the ENSO events, was the most vital determinant of the climate of the Amazon [50]. Smith et al. investigated the variance in leaf area with seasonality and drought using a monthly field LiDAR survey over 4 years and found that the El Niño event strengthened the leaf area seasonal patterns rather than disrupted them [51]. Relative studies are usually focused on specific vegetation regions to obtain a detailed analysis of the influence of climatic oscillation on the growth or carbon absorption of forests based on reasonable field measurements [52, 53]. However, as the interaction between climate variance and forest growth is considerably complex, which not only involves precipitation, temperature and vegetation status, but also includes many other factors, such as the soil nutrients [54, 55], CO_2_ concentration [56, 57] and human activity [58], analyzing this question from a NPP-driven perspective with the help of a big-data approach can help us to obtain more general conclusions on the global level. While this approach can help us accomplish research on a broader spatial and temporal scale, the potential ecological mechanism needs further study combined with specific analysis and detailed field measurements.

## Conclusions

In this article, we have developed a method to analyze the spatiotemporal patterns of global forest NPP and its relationship to diverse impact factors during 2004–2013 from a NPP-category perspective using the big data platform GEE. The forest areas are categorized with the SOM algorithm based on the NPP time series in 10 years to consider their carbon absorption behaviors. Each NPP category comprises various land cover types and climates, so this study can provide an analysis of the global forest NPP from different perspectives than previous studies that analyzing with separate climate and tree categories. Interesting phenomena are found with reasonable indications. With the NPP level changing from low to high, the percentage of warm and wet climate zones increase gradually, while forest components change from low tree to tall tree, from sparse forest to dense forest, and from needle-leaved to broad-leaved three times repeatedly. This nested pattern demonstrates that, compared with the forest types, the influence of climate is stronger on the NPP levels. Forests with low-level NPP are mainly located in the areas of relative cold and dry climates and the forest greenness is positive correlated with precipitation and temperature. As for high-NPP categories, NDVI has a strong positive correlation with FPAR, but weak and even negative correlation with temperature and rainfall. Compared with temperature, precipitation is more likely to show negative correlations with the variation of NDVI for high NPP level forests, and this may attribute to that forests are primarily radiation-limited in tropics. Low-NPP categories show increased precipitation and NPP in La Niña, while high-NPP categories show increased NPP at the onset of the El Niño event and decreased NPP soon afterwards when the warm phase of the ENSO wears off. The role of climate factors for forest greenness and forest carbon absorption are not identical, which implies that forests respond to climate oscillation by changing both photosynthesis and respiration behaviors. The influence of the ENSO on forests correspondingly changes with the NPP levels, which infers that the pattern of climate oscillation and forest growth conditions have some degree of synchronization.

## Supporting information

S1 Fig The result of SOM clustering of NPP during 2001–2013. (PDF)

S2 Fig The monthly FPAR and percentage of FPAR derived from back-up algorithm. (PDF)

S3 Fig The yearly NDVI time series of nine NPP-related forest categories during 2004–2013. (PDF)

S4 Fig The percentage of fill value of yearly NPP during 2004–2013 and monthly NDVI in 2004 of nine NPP categories. (PDF)

S5 Fig The associated MODIS IGBP (International Geosphere-Biosphere Program) land cover types for the NPP-related forest categories. (PDF)

S6 Fig The monthly NDVI and percentage of NDVI>0.8 of nine NPP categories in 2004. (PDF)

S7 Fig Correlations between yearly NPP and impact factors. (PDF)
