# Supplementary material for: Understanding spatiotemporal patterns of global forest NPP using a data-driven method based on GEE
Source: PLoS One. 2020 Mar 10;15(3):e0230098. doi: 10.1371/journal.pone.0230098 (PMC7064189; doi:10.1371/journal.pone.0230098)
Supplement: S1 Fig — (PDF) [file pone.0230098.s001.pdf]

S1 Figure. The result of SOM clustering of NPP during 2004-2013. (a) is the visualization of the SOM of NPP. U-matrix on top left, then component planes and map unit labels on bottom right. The U-matrix indicates distances between neighboring units, and its high values indicate a cluster border, while low values always correspond to clusters themselves. The figure of labels shows the categories represented by each unit. (b) shows the projection of the NPP data to the subspace spanned by its three greatest eigenvectors calculated by principal component analysis (PCA). The nine NPP categories have been plotted in different colors.

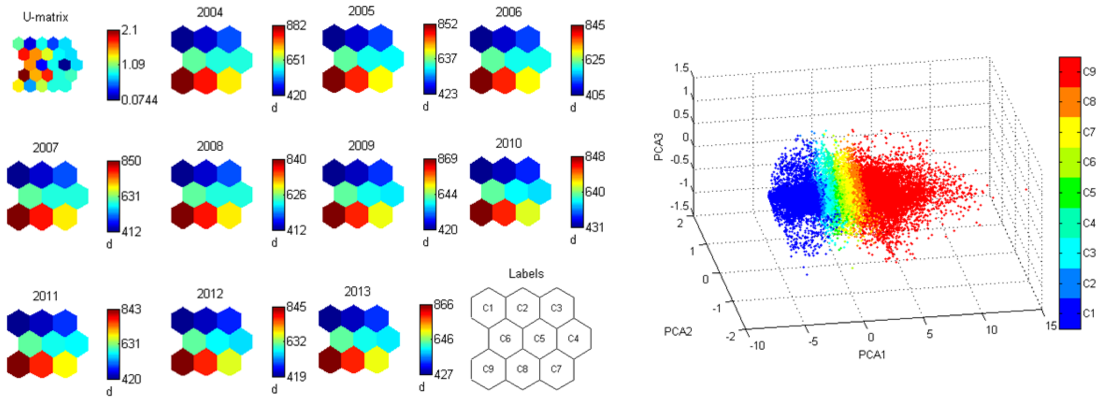

(a) (b)
