# Supplementary material for: Understanding spatiotemporal patterns of global forest NPP using a data-driven method based on GEE
Source: PLoS One. 2020 Mar 10;15(3):e0230098. doi: 10.1371/journal.pone.0230098 (PMC7064189; doi:10.1371/journal.pone.0230098)

S2 Figure. The monthly FPAR and percentage of FPAR derived from back-up algorithm. The FPARs of June to September are relative high (58%-63%), while their percentage of back-up algorithm retrievals are relative low (0-2%). On the contrary, the percentages of back-up algorithm retrievals of January to March and November to December are relative high (14%-28%) and their FPARs are relative low (49%-55%). The average percentage of back-up algorithm retrievals is about 11% for the nine categories.

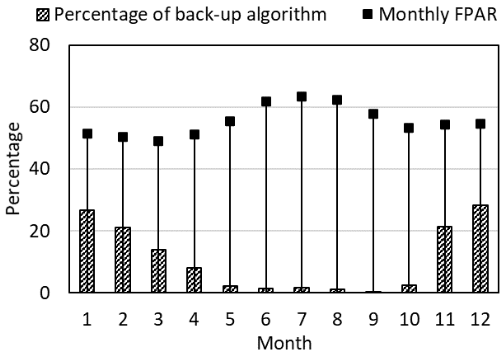

Supplement: S2 Fig — (PDF) [file pone.0230098.s002.pdf]
