# Supplementary material for: Understanding spatiotemporal patterns of global forest NPP using a data-driven method based on GEE
Source: PLoS One. 2020 Mar 10;15(3):e0230098. doi: 10.1371/journal.pone.0230098 (PMC7064189; doi:10.1371/journal.pone.0230098)

S3 Figure. The yearly NDVI time series of nine NPP-related forest categories during 2004-2013. The NDVI of C7-9 increased slightly in 2010. Comparing with the time series of yearly NPP (Fig 1) which shows that the NPP of C7-9 declines in 2010, we can infer that the role of climate factors in the variance of NDVI and NPP may not be entirely the same.

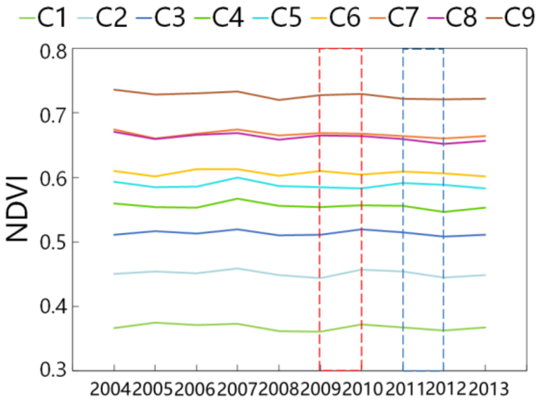

Supplement: S3 Fig — (PDF) [file pone.0230098.s003.pdf]
