# Supplementary material for: Understanding spatiotemporal patterns of global forest NPP using a data-driven method based on GEE
Source: PLoS One. 2020 Mar 10;15(3):e0230098. doi: 10.1371/journal.pone.0230098 (PMC7064189; doi:10.1371/journal.pone.0230098)

S4 Figure. The percentage of fill value of yearly NPP during 2004-2013 and monthly NDVI in 2004 of nine NPP categories. C1 has the most percentage of fill value of yearly NPP, which follows with C2, and C3 only has few bad quality data in 2004, while C4-C9 are all good quality data. In the algorithm of MODIS yearly NPP, the input 8-day or daily data with poor quality are removed and replaced by the interpolated values basing on good quality data. As part of forests of C1 and C2 distribute in the relative high latitude, where may be no good quality data all year round, hence C1 and C2 have more percentage of fill value. As the percentage of fill value of 2004 is highest, the quality of monthly NDVI of the nine categories in this year were analyzed. C9 has the highest percentage of fill value (14%), which followed with C7 (12%) and C8 (10%) and the average percentage of fill value of these nine NPP categories is 9%. As the NDVI data are calculated with MODIS surface reflectance data, it can generally reflect the cloud cover condition of these nine categories.

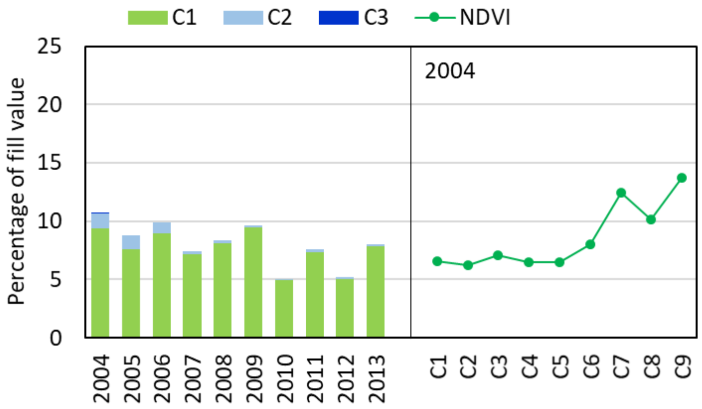

Supplement: S4 Fig — (PDF) [file pone.0230098.s004.pdf]
