# Supplementary material for: Understanding spatiotemporal patterns of global forest NPP using a data-driven method based on GEE
Source: PLoS One. 2020 Mar 10;15(3):e0230098. doi: 10.1371/journal.pone.0230098 (PMC7064189; doi:10.1371/journal.pone.0230098)

S5 Figure. The associated MODIS IGBP (International Geosphere-Biosphere Program) land cover types for the NPP-related forest categories. Each NPP category contains various IGBP land cover types and with the increase of NPP levels, the proportion of forests presents an overall tendency of increasing, while the proportion of herbs displays a decrease tendency in general. This indicates that although high-NPP categories tend to have higher proportion of forest land cover types, the NPP-related categories are not corresponding to specific land cover types.

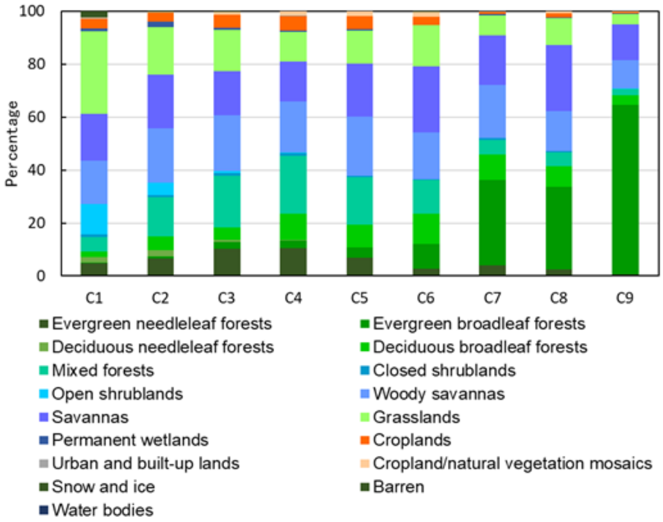

Supplement: S5 Fig — (PDF) [file pone.0230098.s005.pdf]
