# Supplementary material for: Understanding spatiotemporal patterns of global forest NPP using a data-driven method based on GEE
Source: PLoS One. 2020 Mar 10;15(3):e0230098. doi: 10.1371/journal.pone.0230098 (PMC7064189; doi:10.1371/journal.pone.0230098)

S6 Figure. The monthly NDVI and percentage of NDVI>0.8 of nine NPP categories in 2004. The average percentage of NDVI>0.8 for the nine categories is 8% and in line with NDVI and NPP, C9 has the highest percentage (23%) of high NDVI (>0.8). According to previous studies, NDVI reported to be insensitive measure of vegetation amount when greater than 0.8 which caused by the saturation of chlorophyll absorption of red band. The NDVI saturation may take more uncertainties for high-NPP categories.

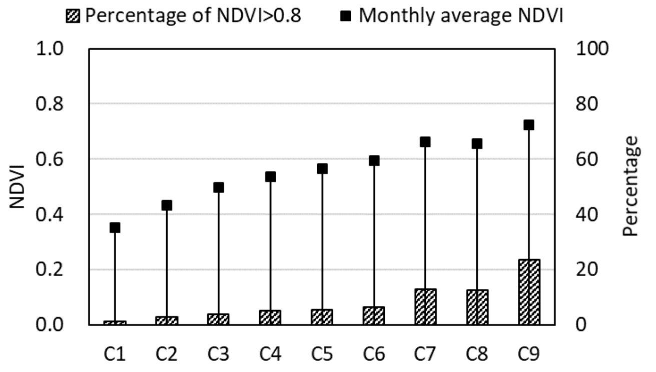

Supplement: S6 Fig — (PDF) [file pone.0230098.s006.pdf]
