# Supplementary material for: Understanding spatiotemporal patterns of global forest NPP using a data-driven method based on GEE
Source: PLoS One. 2020 Mar 10;15(3):e0230098. doi: 10.1371/journal.pone.0230098 (PMC7064189; doi:10.1371/journal.pone.0230098)

S7 Figure. Correlations between yearly NPP and impact factors. For low-NPP categories which reach their peaks in 2010, C1 and C2 have positive correlation between NPP and impact factors, while for high-NPP categories which reach their peaks in 2009, NPP of C7-9 shows weak or negative correlation with precipitation and temperature but exhibits positive correlation with NDVI and FPAR. For C4-6, NPP shows weak or negative correlation with impact factors, which may attribute to the relative slight annual variance. The dispersed distributions from 50°N to 50°S of C4-6 may conceal the regional variation patterns through the average process. Forests of C3 mainly distribute in the transition area of C1-2 and C4-6, and its NPP shows positive correlation with precipitation and weak correlation with other impact factors.

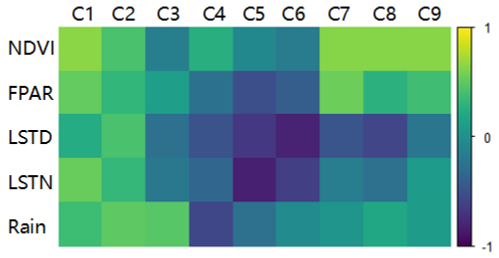

Supplement: S7 Fig — (PDF) [file pone.0230098.s007.pdf]
